# Comment on “Infection outbreaks caused by Carbapenemase-producing Enterobacterales: An epidemiological surveillance study in public hospitals in Chile, 2017–2024”

**DOI:** 10.1016/j.gloepi.2026.100279

**Published:** 2026-07-28

**Authors:** Dilip Kumar Kakru, Hima Bindu Mantravadi, Pankaj Nainwal, Apoorva Makan, Fayaz Ahamed

**Affiliations:** a Department of Microbiology, School of Medical Sciences and Research, Sharda University, Greater Noida, India; b Department of Microbiology, Malla Reddy Instiute of Medical Sciences, Malla Reddy Vishwavidyapeeth, Suraram, Hyderabad 500055, Telangana, India; c Faculty of Pharmaceutical Sciences, Graphic Era Hill University, Dehradun, India; d Centre for Promotion of Research, Graphic Era Deemed University, Dehradun, India; e Dr. D. Y. Patil Medical College, Hospital and Research Centre, Dr. D. Y. Patil Vidyapeeth (Deemed-to-be-University), Pimpri, Pune 411018, Maharashtra, India; f Saveetha Medical College and Hospital, Saveetha Institute of Medical and Technical Sciences, Saveetha University, Chennai, India

Dear Editor,

Lara et al. recently described carbapenemase-producing Enterobacterales (CPE) outbreaks in Chilean public hospitals across pre-pandemic, pandemic, and post-pandemic periods [1]. Their nationwide surveillance offers valuable insight into an evolving antimicrobial-resistance threat. However, several statistical and ascertainment issues in Table 1 require clarification because they directly affect interpretation of the reported temporal changes.

First, the effect estimates labelled "Difference B-A" and "Difference C-A" do not correspond to the displayed medians. Outbreak-size medians were 4, 7, and 4 cases, so the raw differences are 3 and 0 cases, yet Table 1 reports 7 and 4. Duration medians were 75, 47, and 10 days, so the corresponding differences are -28 and -65 days, yet the table reports 45.5 and 9. The estimand may be different from a raw median difference, but the Methods describe bootstrap estimation for differences between medians. The statistic, subtraction direction, confidence-interval procedure, table labels, and associated p values should therefore be verified. STROBE recommends transparent reporting of estimates and their precision so readers can reconstruct the analysis and judge its validity [2].

Second, several sparse proportion contrasts show internally difficult or non-bounded intervals. For example, the post-pandemic versus pre-pandemic difference in attributable deaths is reported as 100 percentage points with a 95% confidence interval of 100 to 100 and p = 0.5, while the corresponding interval for associated deaths extends to 110 percentage points. Although simple asymptotic intervals may cross logical bounds, this behaviour, combined with discordance between intervals and p values, complicates interpretation. Score-based methods for independent proportions provide better coverage and avoid several aberrations of elementary intervals [3]. Reporting the precise method, effect direction, and unrounded estimates, or using exact-compatible approaches for very sparse cells, would improve reliability.

Third, the attack rates appear to be infection-based rather than transmission-based. The denominator comprised contacts in affected units, but colonisation screening was not assessed and only confirmed infections were counted. A population CPE programme using standardised active screening and genomic epidemiology found that 60% of detected CPE episodes represented colonisation and that enhanced surveillance increased case ascertainment without a corresponding rise in clinical infection [4]. Changes in occupancy, sampling intensity, and access to phenotypic, PCR, or genomic methods during 2017–2024 could therefore influence apparent outbreak frequency, duration, and attack rate. Stratification by hospital and diagnostic approach, together with sensitivity analyses restricted to comparable surveillance practices, would strengthen temporal comparisons.

This study addresses important national surveillance data. Correcting or explaining the effect estimates, clarifying the interval methods, and distinguishing observed infection outbreaks from underlying CPE transmission would strengthen its conclusions. Providing the analytic code and a corrected Table 1 would also allow readers to assess whether the central findings remain unchanged.

## Declaration of AI-assisted editing

AI tools, including ChatGPT and Grammarly, were used exclusively for refining the grammar and structure of the manuscript. The interpretative content, conceptual framework, and clinical perspectives were developed entirely by the authors. The final manuscript reflects the authors' independent academic contributions, and the authors take full responsibility for its content.

## CRediT authorship contribution statement

**Dilip Kumar Kakru:** Formal analysis, Methodology, Resources, Software. **Hima Bindu Mantravadi:** Validation, Visualization, Writing – original draft, Writing – review & editing. **Pankaj Nainwal:** Funding acquisition, Investigation, Methodology. **Apoorva Makan:** Resources, Software, Supervision. **Fayaz Ahamed:** Data curation, Formal analysis, Funding acquisition.

## Funding

None.

## Declaration of competing interest

The authors declare that they have no known competing financial interests or personal relationships that could have appeared to influence the work reported in this manuscript.
